# A unique scaphocapitate fracture syndrome in an adolescent: a case report and a review of literature from the last decade

**DOI:** 10.1093/jscr/rjac312

**Published:** 2022-07-04

**Authors:** Hatan Mortada, Mohammed Riyadh AlKhudhair, Ehab F Alsaygh, Abdulaziz S AlModumeegh, Abdullah Kattan

**Affiliations:** Division of Plastic Surgery, Department of Surgery, King Saud University Medical City, King Saud University and Department of Plastic Surgery & Burn Unit, King Saud Medical City, Riyadh, Saudi Arabia; Medical College Al-Imam Muhammad Ibn Saud Islamic University, Riyadh, Saudi Arabia; College of Medicine, Taibah University, Almadinah Almunawwarah, Saudi Arabia; Plastic Surgery Department, Medical College Al-Imam Muhammad Ibn Saud Islamic University, Riyadh, Saudi Arabia; Division of Plastic Surgery, Department of Surgery, College of Medicine, King Saud University, Riyadh, Saudi Arabia

**Keywords:** Scaphocapitate syndrome, carpal tunnel, fracture, scaphoid, capitate, fenton syndrome

## Abstract

Scaphocaptiate fracture syndrome is a unique condition and is challenging to manage. This rare fracture develops after high-energy wrist trauma. We reported a patient with a history of falling on an outstretched hand. A 15-year-old boy had scaphocapitate fracture syndrome, which included a displaced fracture of the capitate, an avulsion fracture at the distal tubercle of the scaphoid bone with an extension to the articular surface, and a perilunate fracture. A few months after open reduction and internal fixation were performed, the patient had a satisfying outcome. In addition, we have conducted a review of the literature regarding the mechanism of injury, diagnosis and outcome of published cases in correlation to our discussed case.

## INTRODUCTION

Fracture dislocations of the carpal bones represent a wide range of complex wrist injuries. The scaphocapitate fracture is a rare injury pattern that occurs due to high-force trauma to the carpus [1]. Scaphocapitate fractures are scaphoid and capitate fractures with rotation of the capitate’s proximal component through 90 or 180 degrees [2]. The uncommon incidence of this syndrome may explain the late diagnosis and maltreatment of such cases [3]. Avascular necrosis (AVN) of the capitate head, posttraumatic arthritis and nonunion are all outcomes of late intervention for a scaphocapitate fracture [4]. We report the first case from Saudi Arabia of scaphocapitate syndrome, emphasizing the mechanism of injury, treatment modality, outcome and result. We also reviewed pertinent literature in relation to this peculiar injury. All of the cases mentioned are characterized as a variant of scaphocapitate fracture syndrome.

## CASE REPORT

**Figure 1:**
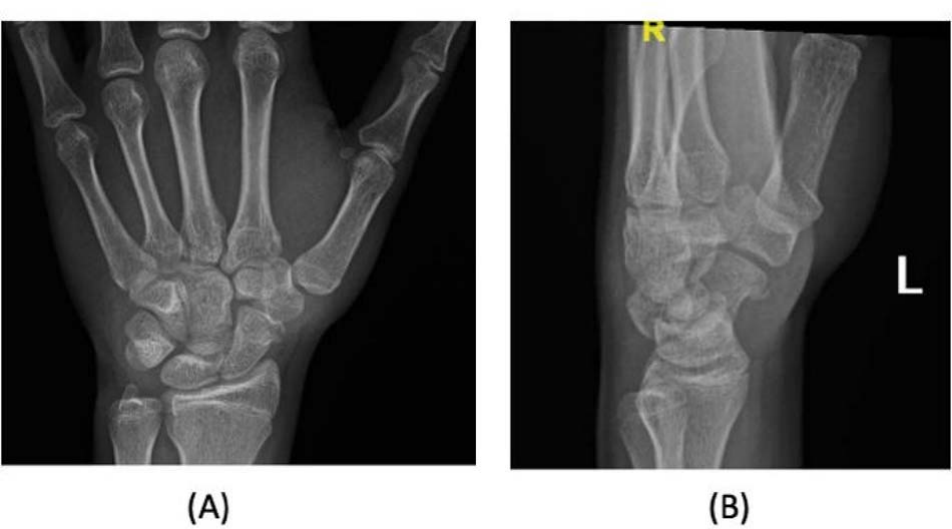
Initial injury posteroanterior and lateral radiograph demonstrating left wrist.

**Figure 2:**
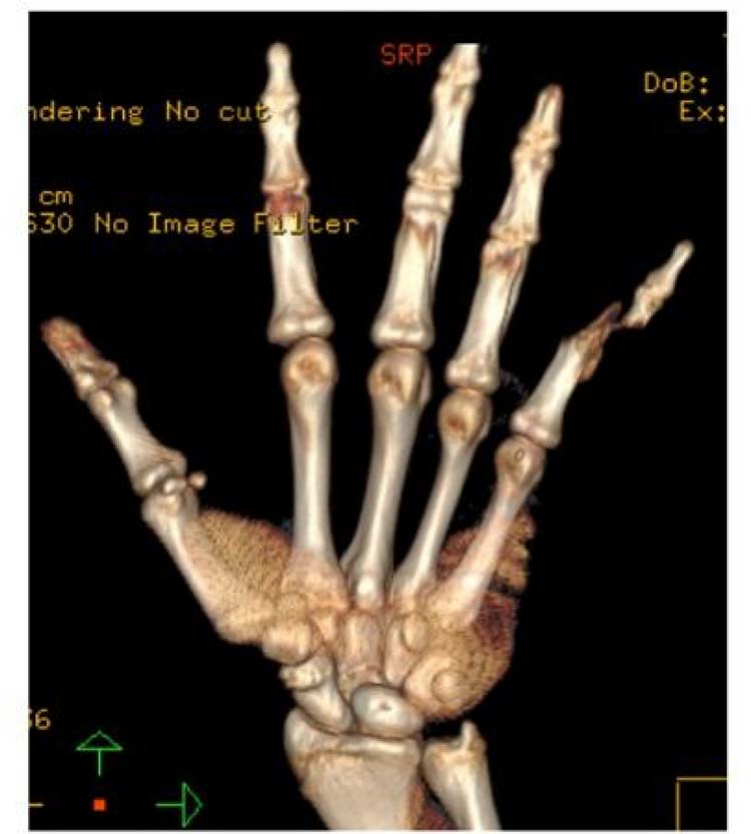
CT scan of the left wrist.

**Figure 3:**
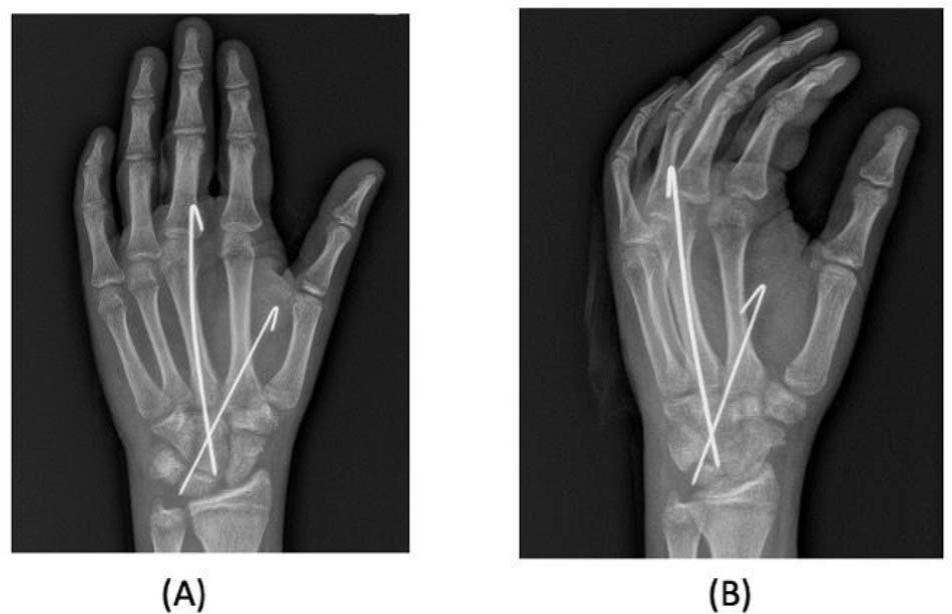
Posteroanterior and oblique radiographs taken intraoperatively after fixation, demonstrating proper alignment of hardware.

**Figure 4:**
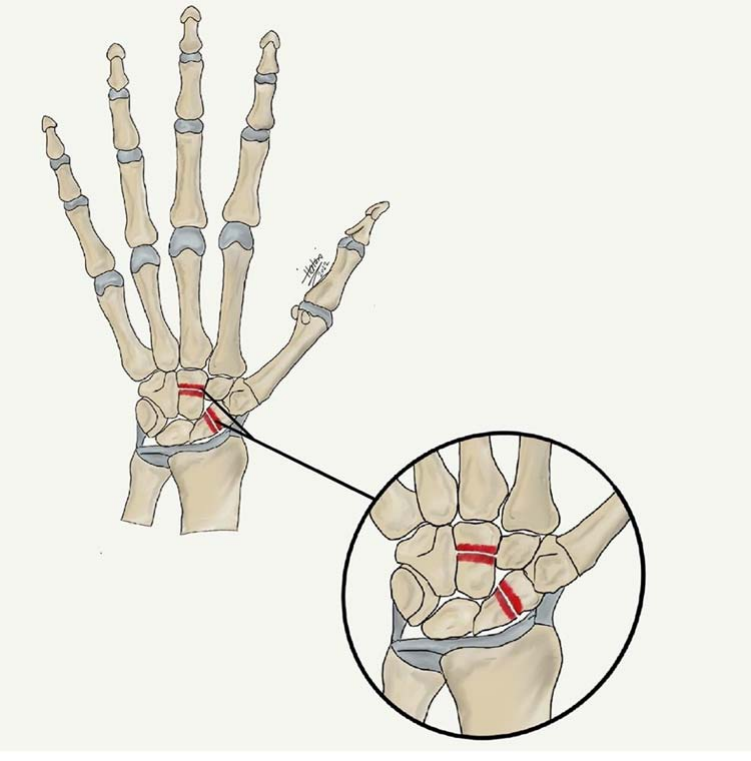
A medical illustration demonstrates scaphocapitate fracture.

A 15-year-old boy, right handed, was not known to have any chronic medical illnesses. He presented to the emergency department (ED) with a history of severe left-wrist pain and swelling after falling from a height of 5–6 meters on an outstretched hand with the wrist in extension three hours prior to the ED presentation. The pain is mainly located in the left wrist’s dorsum and distal part of the forearm. Increasing pain with movement and relived with immobility. On examination, a normal-appearing wrist. No open wounds or lacerations, nor abrasions. There is no obvious deformity. There was mild swelling in the wrist. Tenderness over the dorsal radial wrist, including the anatomical snuff box. The range of motion was restricted due to pain. The neurovascular examination was normal. A plain radiograph in the ED showed a scaphoid tubercle fracture and a capital bone fracture. Initial radiographs are shown in Fig. 1). The patient was taken for computerized tomography (CT) to better define the extent of the injury (Fig. 2). A CT scan showed a displaced fracture of the capitate carpal bone, with an avulsion fracture at the distal tubercle of the scaphoid carpal bone, with extension to the articular surface. In addition to perilunate fracture, the rest of the carpal bone’s alignment is maintained. The patient was shifted to the operating room for open reduction and k-wire fixation of the scaphoid and capitate fracture of the left hand. Under general anesthesia, a tourniquet was applied. The incision was over the dorsal aspect of the left wrist, proceeding with the wrist capsule opening. The fracture was fixated with two Kirschner wires. Alignment was assured intraoperatively with an x-ray (Fig. 3). Plain radiographs post-fixation showed intact alignment. Postoperatively, the wrist was immobilized in a long-arm splint. The Kirschner wires and the protected splint were removed at 5 and 8 weeks, respectively. Immediately afterward, physiotherapy started. A few months following surgery, the site of the fracture healed radiologically. The patient had no pain, and the range of motion was acceptable after a period of physiotherapy. Figure 4 demonstrates a medical illustration of a scaphocapitate fracture.

## DISCUSSION

Scaphocapitate fracture syndrome involves fractures involving the transscaphoid, transcapitate and perilunate fracture dislocation [1]. It was first described by Fenton in 1965 and is recognized as a distinct type of instability of the perilunate. Any injury will disrupt both the scaphoid and capitate bones, which creates a greater arc pattern seen in carpal bone injuries [2]. The exact incidence of scaphocapitate fracture syndrome is unknown. Capitate fractures represent 1.3% of carpal bone fractures, of which 0.6% are scaphocapitate fracture syndrome, 0.4% are capitate fractures associated with perilunate fracture-dislocation injury and 0.3% are isolated capitate fractures [5]. Herein, we present a case of a young patient who presented with a displaced fracture of the capitate carpal bone and an avulsion fracture at the distal tubercle of the scaphoid carpal bone with extension to the articular surface and a perilunate fracture. The concordance of disruption to the perilunate with carpal fractures and the occurrence of scaphocapitate syndrome is common [6]. High-force trauma to the carpus may lead to rupture of the ligaments of the carpus, which may disrupt the integrity of the lunate ligaments when the dorsal capsule and palmar radiolunate ligaments are torn, leading to lunate dislocation [7]. This is in alignment with the etiology of the patient’s injury discussed. Since its original description, approximately 40 cases have been reported in the international literature [8]. This present case is considered the first in the Gulf region and Saudi Arabia. A literature review was conducted (Table 1; [3, 8–12]). We have found that all the cases occurred in men with an average age of 25 years (the range was 12–44 years old). This was the same as what Milliez et al. found [13].

**Table 1:** A review of the literature on scaphocapitate fracture

First author	Study type	Sample size	Gender	Age (years)	Mechanism of injury	Location of fracture	Diagnosis	Outcome
Kim [1]	Case report	1	Male	30	Motor vehicle accident	Anterior dislocation of the scaphoid and lunate, and a comminuted fracture of the capitate with volar displacement of the proximal fragment	Scaphocapitate fracture syndrome	At 12 months, follow-up radiographs revealed bone union and no AVN of the scaphoid or capitate. Complete recovery
Hamdi [9]	Case report	1	Male	22	Falling on outstretched left hand	Transverse fracture of scaphoid associated with proximal pole capitate fracture	Scaphocapitate syndrome	After a 10-month follow-up period, the left wrist was painless, and the grip strength and the range of motion were conserved. X-ray showed the healing of both fractures without evidence of AVN
Fukushi [10]	Case report	1	Male	44	Fall form height	Nonunions at the middle of the capitate and distal third of the scaphoid	Simultaneous nonunion of the scaphoid and capitate	After 1 year, the wrist was painless, and radiography showed healing of both nonunions, without evidence of arthritic changes
Schliemann [8]	Case report	1	Male	19	Falling on left wrist	Lesion of the scaphoid waist and a fracture of the capitate with rotation and a palmar dislocation of the proximal pole	Scaphocapitate fracture	A CT scan at follow-up 14 months after surgery showed a complete consolidation of the fractures with no signs of AVN neither of the scaphoid nor the capitate
Burke [11]	Case report	1	Male	28	Direct trauma	Transstyloid, trans scaphoid, transcapitate fracture with significant rotation of the proximal fragment of the capitate	Transstyloid, transscaphoid, transcapitate fractures	The fracture sites had radiologically healed. The patient was pain free and had a full range of movement after a course of physiotherapy
Afshar [12]	Case Report	1	Male	25	Falling on outstretched hand	Trans-scaphoid fracture-dislocation in both wrist	Bilateral scaphocapitate fracture syndrome	Results of a 5-year follow-up showed satisfactory wrist movements. Wrist arthritis or capitate AVN were not occurred in radiography results
Sawant [3]	Case report	1	Male	12	The dorsum of the right wrist was struck by the rim of the motorcycle headlight	The capitate had fractured through the neck. Its proximal fragment was devoid of any soft tissue attachments and had turned through 180° on its transverse axis	Scaphocapitate syndrome	At the 3-year follow-up examination the wrist was completely asymptomatic. Grip strength was normal. There were no clinical or radiologic signs of AVN

The mechanism of injury for this type of fracture remains contentious in seven cases. Four of them were due to falling into outstretched hands, which is the most common mechanism of injury leading to this type of fracture [9]. The etiology is also consistent with our case. When it comes to diagnosing scaphocapitate fractures, they are frequently missed. As a result of this delay in diagnosis, the management may be delayed and become more complex, leading to a higher rate of complications, such as AVN , posttraumatic carpal arthritis and carpal collapse [6]. Consequently, our patient had apparent fractures in the initial x-rays taken by the ED. Even early treatment of scaphocapitate fracture syndrome is challenging due to damage to soft tissue, bony and cartilaginous structures [13].

Dislocation in scaphocapitate fracture syndrome can be reduced spontaneously with proximal segment inversion of the capitate bone [1]. Seven cases were managed by open reduction and internal fixation, and this management approach was consistent with our case, as the patient was managed with k-wire fixation and immobilization. However, patients who had nondisplaced fractures were managed conservatively. On the other hand, Schädel-Höpfner et al. found that patients who were managed operatively returned earlier to work and noticed improved overall hand functional status [14].

Eventually, both fractures healed well radiologically without AVN or nonunion. However, due to the short follow-up, the longer outcome and complications were not possible to assess. In conclusion, scaphocapitate fracture is a rare and complex injury with challenging diagnosis and management. This study presented a unique case of scaphocapitate fracture involving a scaphoid fracture and a capitate fracture. Early and accurate diagnosis is essential for proceeding with the appropriate management. Proper examination and accurate radiographic investigation are necessary. Open reduction and internal fixation are the management options of choice to prevent complications. We believe reducing the fragment into the original position and fixation of the fractures, as in our patient, is an excellent choice and maybe adequate to accomplish bone union.
